# Long-stay ICU patients with frailty: mortality and recovery outcomes at 6 months

**DOI:** 10.1186/s13613-024-01261-x

**Published:** 2024-02-24

**Authors:** Hannah Wozniak, Tal Sarah Beckmann, Andre Dos Santos Rocha, Jérôme Pugin, Claudia-Paula Heidegger, Sara Cereghetti

**Affiliations:** 1 Division of Critical Care, Department of Anesthesiology, Pharmacology and Intensive Care, Geneva University Hospitals, Geneva, Switzerland; 2 Division of Critical Care Medicine, University of Toronto, Toronto, Canada; 3 Division of Anesthesiology, Department of Anesthesiology, Pharmacology and Intensive Care, Geneva University Hospitals, Geneva, Switzerland

**Keywords:** Frailty, Goals of care, Long-stay ICU patients, Outcomes after critical illness

## Abstract

**Background:**

Prolonged intensive care unit (ICU) stay is associated with physical, cognitive, and psychological disabilities. The impact of baseline frailty on long-stay ICU patients remains uncertain. This study aims to investigate how baseline frailty influences mortality and post-ICU disability 6 months after critical illness in long-stay ICU patients.

**Methods:**

In this retrospective cohort study, we assessed patients hospitalized for ≥ 7 days in the ICU between May 2018 and May 2021, following them for up to 6 months or until death. Based on the Clinical Frailty Scale (CFS) at ICU admissions, patients were categorized as frail (CFS ≥ 5), pre-frail (CFS 3–4) and non-frail (CFS 1–2). Kaplan–Meier curves and a multivariate Cox model were used to examine the association between frailty and mortality. At the 6 month follow-up, we assessed psychological, physical, cognitive outcomes, and health-related quality of life (QoL) using descriptive statistics and linear regressions.

**Results:**

We enrolled 531 patients, of which 178 (33.6%) were frail, 200 (37.6%) pre-frail and 153 (28.8%) non-frail. Frail patients were older, had more comorbidities, and greater disease severity at ICU admission. At 6 months, frail patients presented higher mortality rates than pre-frail and non-frail patients (34.3% (61/178) *vs.* 21% (42/200) *vs*. 13.1% (20/153) respectively, p < 0.01). The rate of withdrawing or withholding of care did not differ significantly between the groups. Compared with CFS 1–2, the adjusted hazard ratios of death at 6 months were 1.7 (95% CI 0.9–2.9) for CFS 3–4 and 2.9 (95% CI 1.7–4.9) for CFS ≥ 5. At 6 months, 192 patients were seen at a follow-up consultation. In multivariate linear regressions, CFS ≥ 5 was associated with poorer physical health-related QoL, but not with poorer mental health-related QoL, compared with CFS 1–2.

**Conclusion:**

Frailty is associated with increased mortality and poorer physical health-related QoL in long-stay ICU patients at 6 months. The admission CFS can help inform patients and families about the complexities of survivorship during a prolonged ICU stay.

**Supplementary Information:**

The online version contains supplementary material available at 10.1186/s13613-024-01261-x.

## Introduction

Prolonged Intensive Care Unit (ICU) stay is associated with increased mortality, which can reach 20–40% at 6 months [1–3]. This association is thought to be multifactorial, depending both on the severity of the illness leading to ICU but also on the baseline vulnerability of these patients [4]. Notably, previous studies have suggested that beyond 10 days in the ICU, antecedent patient characteristics become more predictive of in-hospital mortality than illness severity [5, 6]. Amongst survivors, 30–50% present newly acquired disabilities and long-term sequelae, which may be physical, cognitive or psychological [7–10].

Frailty is a multidimensional syndrome characterized by a lack of physiological reserves, which increases vulnerability and can be quantified using various scales [3, 11, 12]. Although frailty is more prevalent in the elderly community, it is not synonymous with age, and exists among all age groups [10]. The Clinical Frailty Scale (CFS) has been developed in this context, and is a judgement-based frailty assessment tool [13]. It is widely used in the ICU population across all age groups, and can be reliably performed by ICU physicians and researchers [14–16]. Previous studies have reported an association between frailty at ICU admission and both in-hospital mortality, as well as mortality within 6 to 12 months [2, 3, 5, 6, 10, 17]. The proportion of deaths attributed to withholding or withdrawing of care has been described in old ICU patients [18] but is unknown in frail patients with a prolonged ICU stay [19]. In surviving patients, frailty has been associated with poorer long-term outcomes, including diminished quality of life (QoL) and adverse functional outcomes [4, 20, 21]. However, the long-term outcomes of frail patients with a prolonged ICU stay have not been described.

Indeed, long-stay ICU patients are a distinct population from ICU patients with different trajectories [5, 6, 22, 23] and it remains to be determined how pre-admission frailty influence their evolution, mortality and health-related QoL after an ICU stay. In the present study, we analyzed the prevalence of preexisting frailty among long-stay ICU patients, along with their characteristics, and examined their long-term outcomes six months post-ICU admission. We hypothesized that pre-existing frailty is associated with mortality and persistent disabilities 6 months after a prolonged ICU stay. The knowledge of the long-term outcomes of this particular ICU population may help the clinician determine their treatment plan and goals of care.

## Methods

### Study design

We conducted a retrospective monocentric cohort study in the ICU of the Geneva University Hospitals, focusing on patients hospitalized for 7 days or more in the ICU. It is a mixed medical–surgical ICU admitting patients with a wide range of illnesses, including cardiovascular disease, organ transplantation, sepsis and neurologic disease, with about 2′200 admissions per year. Since May 2018, patients hospitalized ≥ 7 days in the ICU are specifically identified by the ICU team as long-stay ICU patients. Six months after admission to the ICU, surviving patients are convened for a post-ICU consultation as the standard of care. If the patient cannot attend the consultation, the reason is documented. For deceased, the date and cause of death is recorded.

During the consultation, a detailed medical history and physical examination are performed. Patients have an evaluation of their mental health, cognitive state, activities of daily living and health-related QoL by answering the Hospital Anxiety and Depression Scale (HADS) [24], Impact of Event Scale Revised (IES-R) [25], Mini Mental State Examination (MMSE) [26], Barthel Index[27], and a short form 12 health survey questionnaire (SF-12) [28]. The use of walking aids as well as the need for nursing home assistance are also evaluated.

### Population selection

We screened all patients identified as long-stay ICU patients between May 1st, 2018 and May 1st, 2021. Exclusion criteria were: age < 18 years old, no documented frailty score, hospitalization for COVID-19 or refusal to participate. The decision not to include COVID-19 patients was made to avoid potential confounding factors from this specific population. Only the first ICU stay of a patient was included in our analysis. The study was conducted in accordance with the Declaration of Helsinki and approved by the Institutional Ethics Committee of Geneva (BASEC 2022–00185).

The assessment of a patient's frailty upon admission to the ICU was conducted by the intensivist in charge at the time of admission using the CFS version 1.0 (Additional file 1: Table S1). This scale includes information obtained from the patient, the primary caregiver, and the patient's medical records. Patients were separated into three groups according to their CFS score [10]: (1) non-frail if CFS < 3, (2) pre-frail if CFS 3–4, and (3) frail if CFS ≥ 5.

### Outcomes

For each patient, demographic data, comorbidities, diagnosis on ICU admission, source of admission, patients' characteristics on ICU admission, severity of illness on ICU admission (Simplified Acute Physiology Score II [SAPS II], Acute Physiology and Chronic Health Evaluation II [APACHE II], and Sequential Organ Failure Assessment [SOFA] score) were collected. During the ICU stay, intubation and duration of mechanical ventilation, initiation of dialysis, initiation of extra-corporeal life support (ECLS), delirium assessed by a positive Confusion Assessment Method for the ICU (CAM-ICU) [29], nosocomial infections, pressure soars, new organ failure were recorded.

At 6 months, data on patient mortality were collected in patients’ chart, including information on whether death followed withholding or withdrawal of care. Withholding was defined as the decision not to initiate nor to increase a treatment intervention in a patient who may potentially benefit from it (i.e. no introduction of vasopressors, no increase in the ongoing vasopressor dose, no intubation, not treating a new complication, etc.) [30]. Withdrawing was defined as the removal of a therapy that had been started in an attempt to sustain life but was not, or no longer, effective or desirable [30]. The censoring date for the survival follow-up was 200 days after ICU admission.

At the 6 months post-ICU consultation, results of the HADS, IES-R, MMSE, SF-12, Barthel index, use of walking aids, and the need for nursing help at home were recorded.

### Statistical analysis

Patients were separated into three groups according to their CFS on ICU admission.

First, a descriptive analysis of patients’ characteristics on ICU admission according to their frailty status was performed. Continuous variables were presented as median with interquartile range (IQR) and categorical variables as number of patients (n) and percentage (%). Chi-squared or Fisher tests were used to detect differences in categorical variables as appropriate and Kruskal–Wallis in continuous variables.

Second, we investigated the association between frailty and mortality using a graphical representation with Kaplan–Meier curves (with log-rank test). Formal testing of the Proportional Hazards (PH) assumption was performed using the Stata command *estat phtest*, and scaled Schoenfeld residuals were generated for graphical analysis of the PH assumption [31]. Finally, to investigate the association between frailty and mortality, we performed a multivariable Cox model, adjusted for a priori selected variables based on a literature review (*i.e.*, SAPS II on ICU admission, Charlson Comorbidity Index, reason for ICU admission and source of admission) [4, 32].

Third, psychological, cognitive and physical outcomes 6 months after ICU admission were compared in survivors according to the frailty status. To further assess the association between frailty on ICU admission and psychological and physical outcomes, multiple linear regressions, adjusted for a priori selected variables were performed (for the physical component of the SF-12: sex, ICU LOS, reason for ICU admission; for psychological and cognitive outcomes: sex, ICU LOS, reason for ICU admission, psychiatric comorbidities, delirium).

To evaluate the bias on the long-term outcomes, considering that only a part of the study sample was able to attend the 6 months follow-up consultation, we performed a sensitivity analysis to compare all the patient characteristics between the initial population and those who were assessed at 6 months.

Due to the low number of missing data, a complete case analysis was performed. For descriptive analyses, missing data were documented in the legend. Two-tailed p-values ≤ 0.05 were considered statistically significant. Statistical analyses were performed using STATA version 16.1 (Stata Corp., College Station, TX, USA, 2007).

## Results

### Study population

Additional file 1: Figure S1 presents the study flowchart. The screening identified 532 long-stay ICU patients for inclusion in the study, with one patient excluded due to missing CFS information. Of the 531 patients included, 29% (153/531) were non-frail, 38% (200/531) pre-frail, and 33% (178/531) frail. Overall, 47% (72/153) of non-frail, 35% (69/200) of pre-frail and 29% (51/178) of frail attended the 6 months follow-up consultation (p < 0.01, Additional file 1: Table S2). Reasons for not coming to the 6 months follow-up consultation were significantly different between groups (p < 0.01, Additional file 1: Table S2), with the main reason for absence in frail patients being death (25% *vs* 32% *vs* 47% in non-frail, pre-frail and frail, respectively).

### Characteristics of long stay ICU patients according to their pre-admission frailty

The characteristics of long stay ICU patients according to their CFS are described in Table 1. Of the 531 included patients, 66.9% (325/531) were male and median age was 59 (IQR, 48–69) years. There was a significant difference in the median age distribution among the various frailty categories (non-frail, 47 (IQR, 36–59); pre-frail, 61 (IQR, 51–71); frail, 64 (IQR, 56–72), p < 0.01). APACHE II scores on ICU admission differed significantly among non-frail (26, IQR 21–30), pre-frail (29, IQR 24–34), and frail (31, IQR 26–36) patients (p < 0.01). Similarly, SAPS II scores on ICU admission varied significantly among non-frail (55, IQR 45–62), pre-frail (61, IQR 51–72), and frail (65, IQR 53–76) patients (p < 0.01).

**Table 1:** Characteristics of Long Stay ICU Patients According to Baseline Frailty

n = 531	CFS 1–2 n = 153	CFS 3–4 n = 200	CFS ≥ 5 n = 178	*p*
Sex, male, n (%)	99 (64.7%)	135 (67.5%)	121 (68%)	0.8
Age (years), median (IQR)	47 (36–59)	61 (51–71)	64 (56–72)	< 0.01
BMI (kg/m^2^), median (IQR)	24.5 (22.5–26.9)	25.9 (23.1–29.3)	25.8 (23.4–29.3)	< 0.01
Source of admission, n (%)				< 0.01
Emergency department	119 (77.8%)	118 (59%)	76 (42.7%)	
Ward	19 (12.4%)	43 (21.5%)	63 (35.4%)	
Other hospital	7 (4.6%)	6 (3%)	12 (6.7%)	
Intermediate care unit	8 (5.2%)	33 (16.5%)	27 (15.2%)	
Admitting diagnosis				< 0.01
Respiratory failure	19 (12.4%)	17 (8.5%)	9 (5.1%)	
Neurological disease	36 (23.5%)	36 (18%)	21 (11.8%)	
Sepsis	2 (1.3%)	8 (4%)	9 (5.1%)	
Cardiovascular disease	19 (12.4%)	35 (17.5%)	42 (23.6%)	
Gastrointestinal disease	45 (29.4%)	45 (22.5%)	45 (25.3%)	
Others	32 (20.9%	59 (29.5%)	52 (29.2%)	
Charlson comorbidity index, median (IQR)	1 (0–2)	3 (1–5)	4 (3–6)	< 0.01
Past medical history of any psychiatric disorder^1^, n (%)	17 (11.1%)	42 (21%)	42 (23.7%)	0.01
APACHEII on ICU admission, median (IQR)	26 (21–30)	29 (24–34)	30.5 (26–36)	< 0.01
SAPSII on ICU admission, median (IQR)	55 (45–62)	61 (51–72)	65 (53–76)	< 0.01
SOFA on ICU admission, median (IQR)	8 (5–11)	9 (7–13)	10 (7–12)	< 0.01
Intubation, n (%)	142 (93.4%)	192 (96%)	156 (87.6%)	< 0.01
Time under MV (days), median (IQR)	9 (5–13)	9 (5–15)	8 (4–13)	0.2
Need for tracheotomy, n (%)	28 (18.4%)	36 (18%)	28 (15.7%)	0.8
Dialysis during ICU stay, n (%)	15 (9.9%)	39 (19.5%)	44 (24.9%)	< 0.01
ECMO during ICU stay, n (%)	15 (9.9%)	27 (13.5%)	16 (9%)	0.4
Nosocomial infection during ICU stay, n (%)	51 (33.3%)	70 (35%)	60 (33.7%)	0.9
Pressure soar during ICU stay, n (%)	37 (24.3%)	58 (29.2%)	73 (41%)	< 0.01
Organ insufficiency during ICU stay, n (%)				< 0.01
Cardiac	11 (7.2%)	17 (8.5)	19 (19.7%)	
Respiratory	30 (19.6%)	44 (22%)	39 (21.9%)	
Renal	37 (24.2%)	56 (28%)	73 (31%)	
Hepatic	11 (7.2%)	13 (6.5%)	14 (7.9%)	
Neurological	4 (2.6%)	11 (5.5%)	11 (6.2%)	
Delirium^2^ during ICU stay, n(%)	41 (27%)	69 (34.5%)	51 (28.7%)	0.3
ICU LOS (days), median (IQR)	14 (11–20)	15 (10–22)	15 (11–19)	0.9
Readmission to ICU after first ICU discharge, n (%)	14 (9.2%)	25 (12.5%)	31 (17.4%)	0.4
Hospital LOS (days), median (IQR)	30 (21–43)	34 (23–58)	37 (22–62)	0.04
Rehabilitation center post ICU, n (%)	80 (58.8%)	96 (56.8%)	74 (52.9%)	0.6
Death at 6 months, n (%)	20 (13.1%)	42 (21%)	61 (34.3%)	< 0.01
Place of death: ICU, n (%)	12 (60%)	22 (52.4%)	27 (44.3%)	0.8
Decision to WD/WH preceding death, n (%)	16 (84.2%)	31 (72.1%)	53 (86.9%)	0.1
Place of the decision to WD or WH, n (%)				< 0.01
WD/WH in the ICU	10 (62.5%)	13 (41.9%)	11 (20.8%)	
WD/WH after ICU discharge	6 (37.5%)	18 (58.1%)	42 (79.3%)	

*Definition of abbreviations: BMI*: body mass index*, MV:* mechanical ventilation, *ECMO:* extracorporeal membrane oxygenation, *ICU:* intensive care unit, *LOS*: length of stay, WD: withdraw, WH: withhold

Results reported as *n* (%) for categorical variables and median [IQR] for continuous variables

^1^depression, anxiety, borderline, bipolar, other

^2^defined by at least one positive CAM-ICU (Confusion Assessment Method for the ICU) during ICU stay

Intubation was required in 93.4% (142/152) non-frail patients, 96% (192/200) pre-frail patients, and 87.6% (156/178) frail patients (p < 0.01). No significant difference was observed between frail and non-frail patients in terms of duration of mechanical ventilation, need for tracheostomy, and ICU and hospital LOS.

### Frailty score and mortality

**Fig. 1:**
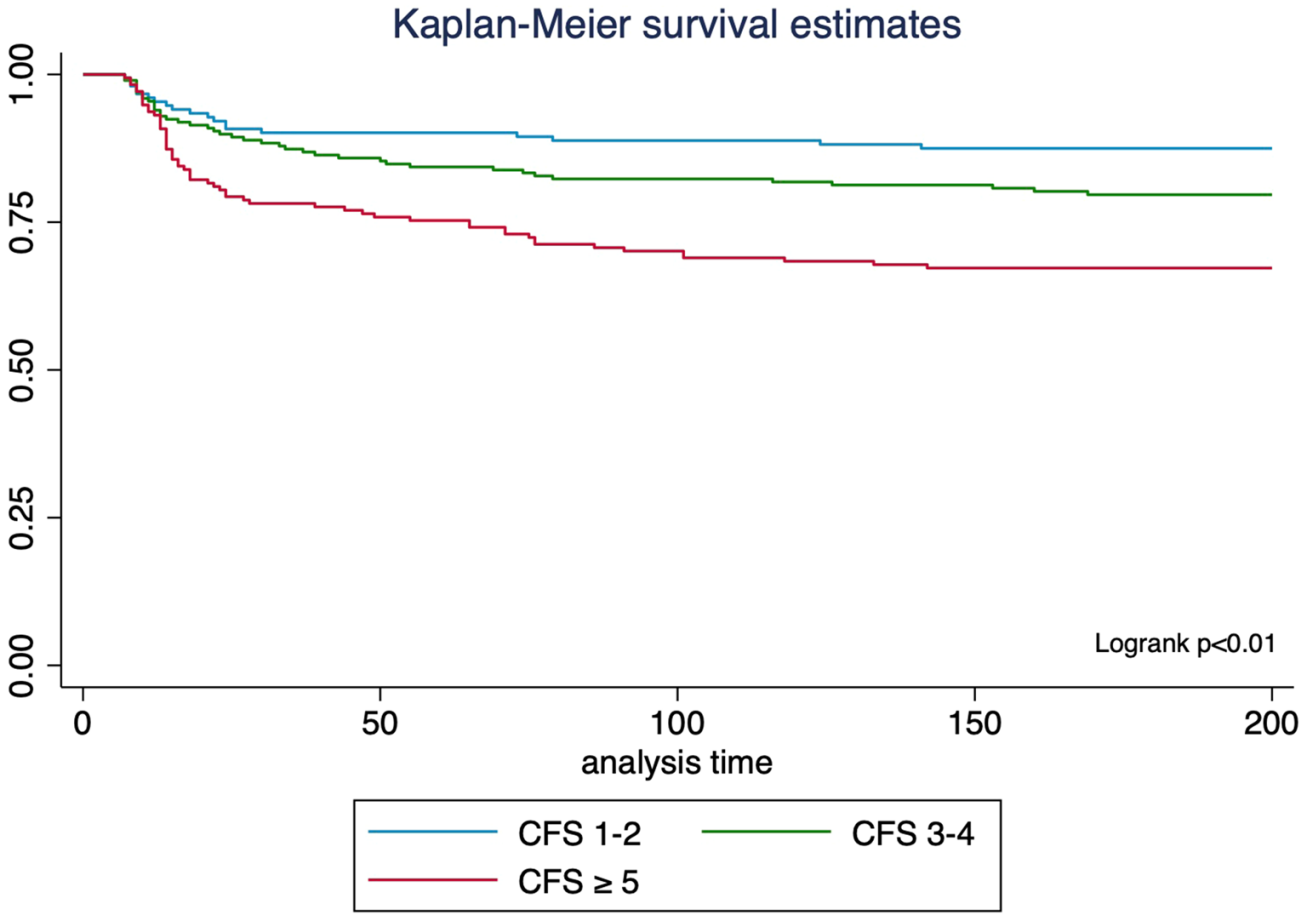
Survival Estimates According to Frailty, Using Kaplan–Meier

Overall, 23.1% (123/531) of ICU long-stayers died within 6 months. Higher mortality at 6 months was observed in frail patients (34%, 61/178) compared to pre-frail (21%, 42/200) and non-frail (13%, 20/152) patients (p < 0.01). The rate of withdrawing or withholding of care did not differ significantly between the groups. The survival estimates over 6 months are represented through the Kaplan–Meier method in Fig. 1. The multivariable Cox model showed an adjusted hazard ratio of death of 1.7 (95% CI 0.9–2.9) for pre-frail patients and 2.9 (95% CI 1.7–4.9) for frail patients, compared to non-frail patients (Table 2).

**Table 2:** Association Between Baseline Frailty and Mortality at 6-Months After ICU Admission

n = 524*	Mortality at 6 months, Hazard Ratio (95% CI)	*p*
Univariate analysis		
CFS 1–2	Reference	
CFS 3–4	1.7 (0.9–2.9)	0.065
CFS ≥ 5	2.9 (1.7–4.9)	< 0.01
Multivariate analysis		
Frailty status		
CFS 1–2	Reference	
CFS 3–4	1.3 (0.7–2.4)	0.3
CFS ≥ 5	2.1 (1.1–3.8)	0.02
Charlson comorbidity index, per point	1.1 (1.06–1.2)	< 0.01
SAPS II on ICU admission, per point	1 (0.9–1.01)	0.8
Reasons for ICU admission		
Respiratory failure	Reference	
Neurological disease	0.7 (0.3–1.4)	0.3
Septic shock	0.7 (0.2–2.4)	0.5
Abdominal disease	0.8 (0.4–1.5)	0.4
Cardiovascular disease	0.5 (0.2–1.2)	0.1
Others	0.8 (0.4–1.6)	0.5
Source of admission		
Emergency department	Reference	
Ward	0.6 (0.4–1)	0.08
Transfer from another hospital	1.5 (0.7–3.1)	0.3
Intermediate care unit	0.7 (0.4–1.3)	0.3

^*^7 patients excluded, missing data on the time of follow-up

Results reported as Hazard Ratio (HR) with 95% Confidence Intervals (95% CI)

### Frailty and long-term outcomes

A sensitivity analysis comparing the patients who attended the 6 months post-ICU consultation and the initial ICU population sample did not show significant differences in the patient characteristics (Additional file 1: Table S3). We therefore assumed that outcomes at 6 months could be extrapolated to the study population without significant sample bias.

**Fig. 2:**
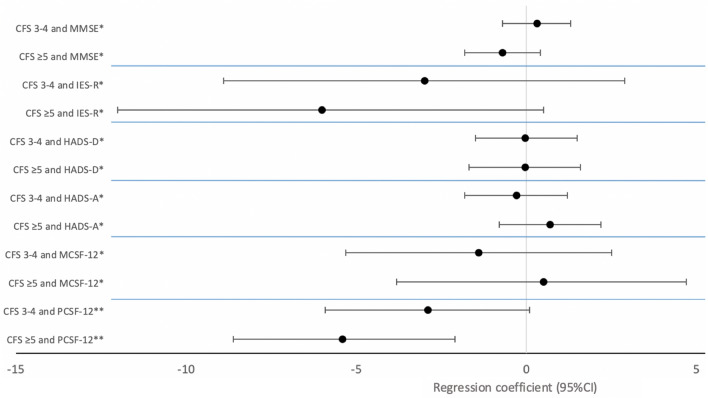
Association of Frailty with Psychological, Cognitive, and Physical Outcomes at 6 Months after ICU Admission in Surviving ICU Patients. Regression coefficient plot with confidence intervals of standard multiple linear regressions. Definition of abbreviations: IES-R: Impact of Event Scale-Revised; HADS: Hospital Anxiety and Depression Scale, HADS-D: refers to the depression components of HADS, HADS-A: refers to the anxiety component of HADS; PCSF-12: physical component of the SF-12; MCSF-12: mental component of the SF-12; MMSE, Mini Mental State Examination. *Adjusted to: sex, ICU length of stay, reason for ICU admission, psychiatric comorbidities, presence of delirium. **Adjusted to: sex, ICU length of stay, reason for ICU admission

At the 6 months consultation, 192 patients were evaluated, out of which 51 were identified as frail prior to ICU admission (Table 3). Frail patients were more likely to need nursing assistance at home (53%) compared to pre-frail (41%) and non-frail (24%) patients (p < 0.01). The physical component of the QoL assessment using the SF-12 questionnaire tended to be lower in frail patients (35 (IQR, 30.8–43.1)) compared to pre-frail (37.1 (IQR, 30.5–43.8)) and non-frail (40.1 (IQR, 32.9–47)) patients (p = 0.058). Uni- and multivariate linear regressions showed a significant association between pre-ICU frailty, and lower physical component of QoL at 6-months (p < 0.01 for both, Fig. 2 and Additional file 1: Table S4). At 6 months, there was no significant difference between the three groups in the univariate and multivariate analysis in terms of cognitive impairment, anxiety, depressive symptoms, PTSD symptoms as well as the psychological component of QoL (Fig. 2 and Additional file 1: Table S4).

**Table 3:** Six-Month Outcomes after ICU Admission in Surviving Patients

n = 192	CFS 1–2 N = 72	CFS 3–4 N = 69	CFS ≥ 5 N = 51	*p*
Physical outcomes at 6 months				
Physically active before ICU, n (%)^1^	49 (73.1%)	33 (50%)	28 (57.1%)	0.02
Walking aids at 6 months, n (%)	8 (11.1%)	24 (34.8%)	11 (21.6%)	< 0.01
Barthel index, median (IQR)^2^	100 (100–100)	100 (92–100)	100 (95–100)	0.05
Psychological outcomes at 6 months				
Any mood disorder, n (%)^3^	34 (52.3%)	27 (45.8%)	21 (46.7%)	0.7
HADS-A, median (IQR)^4^	5 (3–8)	4 (3–6)	5 (3–10)	0.2
Anxiety defined by HADS ≥ 8, n (%)^4^	18 (29%)	11 (19%)	17 (37.8%)	0.1
HADS-D, median (IQR)^4^	4 (2–7)	4 (2–7)	4 (2–8)	0.6
Depression defined by HADS ≥ 8, n (%)^4^	15 (24.2%)	14 (24.1%)	13 (28.9%)	0.8
IES-R score, median (IQR)^5^	16 (7–33)	14 (4–28)	10 (4–28)	0.3
PTSD defined by IES-R ≥ 30, n (%)^5^	21 (31.3%)	16 (25%)	10 (22.2%)	0.5
Cognitive outcome at 6 months				
MMSE (points), median (IQR)^6^	29 (26–30)	29 (27–30)	28 (26–29)	0.2
Health-related quality of life assessment at 6 months				
Mental health-related quality of life (SF-12), median (IQR)^7^	48.5 (40–55.9)	47.7 (38.6–56.6)	47.6 (39.8–56.1)	0.9
Physical health-related quality of life (SF-12), median (IQR)^7^	40.1 (32.9–47)	37.1 (30.5–43.8)	35 (30.8–43.1)	0.058
Resource utilization				
Need of nursing help at home, n (%)	17 (23.6%)	28 (40.6%)	27 (52.9%)	< 0.01
Psychological follow-up, n (%)	16 (22.2%)	14 (20.3%)	6 (11.8%)	0.3
Readmission between hospital leave and consultation, n (%)	17 (23.6%)	23 (33.3%)	17 (33.3%)	0.3

Definition of abbreviations: HADS: Hospital Anxiety and Depression Scale, HADS-A: refers to the anxiety component of HADS, HADS-D: refers to the depression components of HADS, PTSD: post-traumatic stress disorders, IES-R: Impact of Event Scale-Revised, MMSE: Mini-Mental State Examination, SF-12: Short Form Health Survey 12-items

Results reported as *n* (%) for categorical variables and median (IQR) for continuous variables

Missing data: ^1^8 (5 CFS 1–2, 1 CFS 3–4, 2 CFS ≥ 5),^2^13 (5 CFS 1–2, 1 CFS 3–4, 7 CFS ≥ 5), ^3^21 missing (7 CFS 1–2, 8 CFS 3–4, 6 CFS ≥ 5), ^4^26 missing data (10 CFS 1–2, 10 CFS 3–4, 6 CFS ≥ 5), ^5^15 missing data (5 CFS 1–2, 4 CFS 3–4, 6 CFS ≥ 5), ^6^22 missing data (5 CFS 1–2, 9 CFS 3–4, 8 CFS ≥ 5), ^7^20 missing data (7 CFS 1–2, 7 CFS 3–4, 6 CFS ≥ 5)

## Discussion

In the present study, one-third of the patients who stayed ≥ 7 days in the ICU were frail (CFS ≥ 5) on ICU admission. Frail patients are a distinct population, presenting more comorbidities, advanced age, and higher severity of illness upon admission to the ICU. The 6-month post-ICU mortality rate was higher among frail patients, with a similar trend observed in pre-frail patients compared to non-frail individuals. Among patients who survived in the ICU, frail patients had poorer physical health-related QoL at 6 months. However, there was no association between frailty and mental health-related QoL, anxiety or depressive symptoms, symptoms of post-traumatic stress disorders (PTSD) and cognitive outcomes at 6 months.

ICU patients with pre-existing frailty account for up to 30% of admissions and are associated with increased mortality at 6 months post-ICU [3, 4, 10, 20, 33]. However, long-stayers have not been studied specifically, despite representing a distinct population [22, 23]. In addition, the proportion of deaths following withdrawing or withholding of care was usually not specified in the previous studies [20, 30, 34]. Our study focused on long-stay ICU patients and found that frailty at admission was associated with increased mortality, while the decision to withhold or withdraw care preceded 81% of deaths with no significant difference between frail and non-frail patients. This differs from the results of a large multicenter study that found that frailty was associated with the decision to withdraw and withhold life-sustaining therapy in ICU patients over 80 years old [35]. Clinicians often question the appropriateness of intensive therapies, fearing that such treatments may be futile or even harmful to the patient [36, 37]. Our findings suggest that other factors besides frailty contribute to the decision-making. Variations in intensivists' awareness of chances of survival and post-ICU outcomes [23, 38] may have played a role in shaping decisions, as well as age, given our study's inclusion of patients of all ages, differing from the cited study.

Physical, psychological and cognitive sequelae after prolonged ICU stay are common [23]. The results from our study show that pre-admission frailty was associated with poorer physical health-related QoL at 6 months in ICU long-stayers compared to non-frail patients, but not with poorer mental health-related QoL. This is congruent with the results of a study by Brummel et al., who observed the same association at 3 and 12 months after ICU admission, albeit without a specific focus on long-stay ICU patients [4]. This is also reflected in the finding that frail patients in our study more often require nursing assistance at home after their ICU stay. Physicians should discuss goals of care with the patient and their family, considering the potential impact of frailty on post-ICU QoL and mortality.

Although our study found no association between frailty and mental health-related QoL in ICU long-stayers, conflicting findings have been reported on this topic. In a study by Bagshaw et al. an association was found between frailty and worse mental health-related QoL, contrasting with Brummel’s and our study [4, 21]. An association between frailty upon ICU admission and depressive and anxiety symptoms was also reported [20, 21]. Previous research suggests that patients who are already disabled or have a low health-related QoL may be less psychologically affected by further health deterioration than previously healthy patients [20, 39–42]. It is possible that frail patients had a low health-related QoL prior to the ICU stay, which may make them less susceptible to psychological distress from further decline. However, without data on the pre-ICU health-related QoL and disability of frail patients, this hypothesis cannot be confirmed by our study. Further research is needed to better understand the relationship between frailty and mental health-related QoL in this population.

Finally, our study did not observe an association between frailty and increased cognitive dysfunction, which is consistent with Brummel’s study [4]. This is somewhat surprising since frailty, outside the context of critical illness, has been associated with a higher risk of dementia [43]. It is possible that such an association may develop later in our frail patient population [4]. Further studies with longer follow-up periods are needed to better understand the relationship between frailty and cognitive function in this population.

Our study has several limitations. First, 63.8% of the initial population did not have a follow-up consultation at 6 months, primarily due to mortality. This led to a potential risk of selection bias. Nevertheless, a sensitivity analysis confirmed that patient characteristics at the consultation did not significantly differ from those of the initial long-stay ICU population. Second, our study lacks information on the patients' QoL before their stay in the ICU. Thus, we could not differentiate whether the poor health-related physical QoL was due to deterioration or prior disability. Third, although the CFS score is a validated tool that is widely used in ICU studies, there may be some subjectivity [4]. Our ICU team has been trained in the use of this scale which has proven to be reliable in this setting [14, 15]. Fourth, despite the study period encompassing the COVID-19 period, potentially influencing the admission and care of frail patients, our center, as the reference center for ICU admission, maintained consistent practices, minimizing this potential bias. Lastly, the design of our study does not allow us to infer causality between frailty and mortality and the outcomes measured at 6 months, but only to establish an association.

## Conclusion

Our study shows that one-third of patients hospitalized in the ICU for ≥ 7 days were frail prior to their admission. Frailty was independently associated with increased mortality at 6 months. However, frail patients did not have a higher occurrence of death following decisions to withdraw or withhold life sustaining therapies compared to non-frail patients. In surviving patients, being frail is associated with poorer physical health-related QoL at 6 months post-ICU, but not with lower mental health-related QoL. The CFS is a reliable and simple tool for measuring frailty, which can provide insights to patients and families about the challenges of survivorship during a prolonged ICU stay.

### Supplementary Information

**Additional file 1: Table S1.** The Clinical Frailty Scale (CFS)–version 1.0. **Table S2.** Patients follow-up at 6 months according to their frailty score on ICU admission. **Table S3.** Sensitivity analysis: descriptions of long stay ICU patients according to their frailty score on ICU admission, excluding patients who did not come to the ICU consultation. **Table S4.** Association Between Frailty and Psychological, Cognitive, and Physical Outcomes at 6 Months after ICU Admission in Surviving ICU Patients. **Figure S1.** Study Flowchart.

## Data Availability

The datasets used and/or analysed during the current study are available from the corresponding author on reasonable request.

## Abbreviations

ICU: Intensive care unit
CFS: Clinical Frailty Scale
QoL: Quality of Life
HADS: Hospital Anxiety and Depression Scale
IES-R: Impact of Event Scale Revised
MMSE: Mini Mental State Evaluation
SF-12: Short form 12 health survey questionnaire
SAPS II: Simplified Acute Physiology Score II
APACHE II: Acute Physiology and Chronic Health Evaluation II
SOFA: Sequential Organ Failure Assessment score
CAM-ICU: Confusion Assessment Method for the ICU
ECLS: Extra-corporeal life support
PTSD: Post-traumatic stress disorder
WD: Withdrawal
WH: Withholding
