# Supplementary material for: Long-stay ICU patients with frailty: mortality and recovery outcomes at 6 months
Source: Ann Intensive Care. 2024 Feb 24;14:31. doi: 10.1186/s13613-024-01261-x (PMC10894177; doi:10.1186/s13613-024-01261-x)

**Additional file**

**Additional file 1: Table S1. The Clinical Frailty Scale (CFS) – version 1.0**

| **CFS 1** | Very fit – People who are robust, active, energetic and motivated. These people commonly exercise regularly. They are among the fittest for their age. |
| --- | --- |
| **CFS 2** | Well – People who have no active disease symptoms but are less fit than category 1. Often, they exercise or are very active occasionally, e.g. seasonally. |
| **CFS 3** | Managing Well – People whose medical problems are well controlled, but are not regularly active beyond routine walking |
| **CFS 4** | Vulnerable – While not dependent on others for daily help, often symptoms limit activities. A common complaint is being “slowed up”, and/or being tired during the day. |
| **CFS 5** | Mildly Frail – These people often have more evident slowing, and need help in high order IADLs (finances, transportation, heavy housework, medications). Typically, mild frailty progressively impairs shopping and walking outside alone, meal preparation and housework |
| **CFS 6** | Moderately Frail – People need help with all outside activities and with keeping house. Inside, they often have problems with stairs and need help with bathing and might need minimal assistance (cuing, standby) with dressing. |
| **CFS 7** | Severely Frail – Completely dependent for personal care, from whatever cause (physical or cognitive). Even so, they seem stable and not at high risk of dying (within ~ 6 months). |
| **CFS 8** | Very Severely Frail – Completely dependent, approaching the end of life. Typically, they could not recover even from a minor illness. |
| **CFS 9** | Terminally Ill - Approaching the end of life. This category applies to people with a life expectancy <6 months, who are not otherwise evidently frail. |

*Adapted from: Rockwood K, Song X, MacKnight C, Bergman H, Hogan DB, McDowell I, Mitnitski A. A global clinical measure of fitness and frailty in elderly people. CMAJ. 2005 Aug 30;173(5):489-95. doi: 10.1503/cmaj.050051. PMID: 16129869; PMCID: PMC1188185.*

**Additional file 1: Table S2. Patients follow-up at 6 months according to their frailty score on ICU admission**

| **n= 531** | **CFS 1-2**  n=153 | **CFS 3-4**  n=200 | **CFS≥5**  n=178 | *p* |
| --- | --- | --- | --- | --- |
| Consultation 6 months post ICU discharge, n= 192 | 72 (47.1%) | 69 (34.5%) | 51 (28.6%) | <0.01 |
|  |  |  |  |  |
| **Patients who did not come to the 6 months consultation,** n=339 | **Non-frail**  n=81 | **CFS 3-4**  n= 131 | **Frail**  n= 127 | *p* |
| Reason for no consultations:  -patient not living in Geneva anymore  -refusal to participate as felt well  -death  -too sick to come or hospitalised  -patient living in a nursing home  -alive, reason not documented | 34 (42%)  14 (17.3%)  20 (24.7%)  3 (3.7%)  3 (3.7%)  7 (8.6%) | 33 (25.4%)  27 (20.8%)  42 (32.3%)  14 (10.9%)  7 (5.4%)  8 (6.2%) | 12 (9.4%)  31 (24.4%)  60 (47.2%)  12 (9.4%)  6 (4.7%)  6 (4.7%) | <0.01 |

Results reported as *n* (%) for categorical variables

**Additional file 1: Table S3. Sensitivity analysis: descriptions of long stay ICU patients according to their frailty score on ICU admission, excluding patients who did not come to the ICU consultation**

| **n= 192** | **CFS 1-2**  n=72 | **CFS 3-4**  n=69 | **CF≥5**  n=51 | *p* |
| --- | --- | --- | --- | --- |
| Gender, male, n (%) | 46 (63.9%) | 47 (68.1%) | 39 (76.5%) | 0.3 |
| Age, median (IQR) | 46 (33-58) | 59 (50-71) | 64 (53-74) | <0.01 |
| BMI, median (IQR) | 24.7 (23-27) | 26 (23.9-30.4) | 27.2 (24.4-29.4) | 0.02 |
| Country of origin, not swiss, n (%) | 25 (34.7%) | 25 (36.2%) | 25 (50%) | 0.3 |
| Source of admission, n (%)  -emergency  -ward  -other hospital  -intermediate care unit | 53 (73.6%)  11 (15.3%)  2 (2.8%)  6 (8.3%) | 39 (56.5%)  21 (30.4%)  2 (2.9%)  7 (10.1%) | 23 (45.1%)  19 (37.3%)  2 (3.9%)  7 (12.7%) | 0.051 |
| Reason for admission, n(%):  -respiratory insufficiency  -neurological issue  -sepsis  -CV issue  -dig/uro  -other | 6 (8.3%)  20 (27.8%)  1 (2.4%)  7 (9-7%)  18 (25%)  20 (27.8%) | 3 (4.4%)  15 (21.7%)  4 (5.8%)  11 (15.9%)  12 (17.4%)  24 (34.8%) | 3 (5.9%)  6 (11.8%)  4 (7.8%)  10 (19.6%)  13 (25.5%)  15 (29.4%) | 0.3 |
| Charlson comorbidity score, median (IQR) | 1 (0-2) | 3 (1-5) | 4 (3-7) | <0.01 |
| Past medical history of any psychiatric disorder*, n (%) | 6 (8.3%) | 12 (17.4%) | 10 (20%) | 0.1 |
| APACHEII, median (IQR) | 25 (21-30) | 31 (24-36) | 31 (27-36) | <0.01 |
| SAPSII, median (IQR) | 55 (46-62) | 64 (54-75) | 62 (50-76) | <0.01 |
| SOFA, median (IQR) | 8 (5-11) | 10 (7-14) | 11 (7-13) | <0.01 |
| Intubation, n(%) | 65 (90.3%) | 66 (95.7%) | 48 (94.1%) | 0.2 |
| Time under MV, median (IQR) | 9 (5-12) | 9 (5-16) | 6 (4-11) |  |
| Need for tracheotomy, n (%) | 7 (9.9%) | 8 (11.6%) | 6 (11.8%) | 0.9 |
| Need for dialysis, n (%) | 8 (11.1%) | 19 (27.5%) | 15 (30%) | 0.02 |
| Need for ECMO, n (%) | 12 (16.7%) | 12 (17.4%) | 5 (9.8%) | 0.5 |
| Delirium during ICU stay, n(%) | 20 (28.2%) | 31 (44.9%) | 20 (39.2%) | 0.1 |
| ICU LOS, median (IQR) | 13 (10-18) | 15 (10-23) | 14 (10-21) | 0.1 |
| Readmission to ICU after first ICU discharge, n (%) | 3 (4.2%) | 12 (17.4%) | 6 (11.8%) | 0.04 |
| Hospital LOS, median (IQR) | 30 (23-43) | 36 (26-66) | 44 (23-60) | 0.02 |
| Rehabilitation post ICU needed, n (%) | 43 (59.7%) | 41 (59.4%) | 22 (44%) | 0.2 |

*Definition of abbreviations: BMI*: body mass index*, CV:* cardiovascular, *MV:* mechanical ventilation, *ECMO:* extracorporeal membrane oxygenation, *ICU:* intensive care unit, *LOS*: length of stay.

Results reported as *n* (%) for categorical variables and median [IQR] for continuous variables.

^1^depression, anxiety, borderline, bipolar, other

**Additional file 1: Table S4. Association Between Frailty and Psychological, Cognitive, and Physical Outcomes at 6 Months after ICU Admission in Surviving ICU Patients**

|  | **PCSF-12, β-Coefficient (95% CI)** | *p* |
| --- | --- | --- |
| Frailty on ICU admission, n= 173  -CFS 1-2  -CFS 3-4  -CFS ≥5 | Ref  -2.4 (-5.4 – 0.5)  -4.4 (-7.6 – -1.2) | 0.1  <0.01 |
| Frailty on ICU admission*, n= 173  -CFS 1-2  -CFS 3-4  -CFS ≥5 | Ref  -2.9 (-5.9 – 0.1)  -5.4 (-8.6 – -2.1) | 0.06  <0.01 |
|  |  |  |
|  | **MCSF-12, β-Coefficient (95% CI)** |  |
| Frailty on ICU admission, n= 173  -CFS 1-2  -CFS 3-4  -CFS ≥5 | Ref  -0.9 (-4.5 – 2.8)  0.3 (-3.7– 4.2) | 0.6  0.9 |
|  |  |  |
| Frailty on ICU admission**, n= 171  -CFS 1-2  -CFS 3-4  -CFS ≥5 | Ref  -1.4 (-5.3 – 2.5)  0.5 (-3.8 – 4.7) | 0.4  0.8 |
|  | **HADS-A (Anxiety), β-Coefficient (95% CI)** |  |
| Frailty on ICU admission, n= 166  -CFS 1-2  -CFS 3-4  -CFS ≥5 | Ref  -0.5 (-1.9 – 0.9)  0.7 (-0.8 – 2.2) | 0.5  0.4 |
|  |  |  |
| Frailty on ICU admission**, n=164  -CFS 1-2  -CFS 3-4  -CFS ≥5 | Ref  -0.3 (-1.8 – 1.2)  0.7 (-0.9 – 2.2) | 0.7  0.4 |
|  | **HADS-D (Depression), β Coefficient (95% CI)** |  |
| Frailty on ICU admission, n= 166  -CFS 1-2  -CFS 3-4  -CFS ≥5 | Ref  -0.3 (-1.7 – 1.2)  -0.2 (-1.7 – 1.4) | 0.7  0.8 |
|  |  |  |
| Frailty on ICU admission**, n=164  -CFS 1-2  -CFS 3-4  -CFS ≥5 | Ref  -0.03 (-1.5 – 1.5)  -0.04 (-1.7 – 1.6) | 0.9  0.9 |
|  | **IES-R, β-Coefficient (95% CI)** |  |
| Frailty on ICU admission, n= 177  -CFS 1-2  -CFS 3-4  -CFS ≥5 | Ref  -2.4 (-8 – 3.2)  -4.4 (-10.6 – 1.7) | 0.4  0.2 |
|  |  |  |
| Frailty on ICU admission**, n= 175  -CFS 1-2  -CFS 3-4  -CFS ≥5 | Ref  -3 (-8.9 – 2.9)  -6 (-12.4 – 0.5) | 0.3  0.07 |
|  | **MMSE, β-Coefficient (95% CI)** |  |
|  |  |  |
| Frailty on ICU admission, n= 171  -CFS 1-2  -CFS 3-4  -CFS ≥5 | Ref  0.04 (-0.9 – 1)  -0.8 (-1.8 – 0.3) | 0.9  0.1 |
| Frailty on ICU admission**, n= 169  -CFS 1-2  -CFS 3-4  -CFS ≥5 | Ref  0.3 (-0.7 – 1.3)  -0.7 (-1.8 – 0.4) | 0.6  0.2 |

Definition of abbreviations: IES-R: Impact of Event Scale-Revised; HADS: Hospital Anxiety and Depression Scale, HADS-D: refers to the depression components of HADS, HADS-A: refers to the anxiety component of HADS; PCSF-12: physical component of the SF-12; MCSF-12: mental component of the SF-12; MMSE, Mini Mental State Examination.

*After adjustment to: gender, ICU LOS, reason for ICU admission

**After adjustment to: gender, ICU LOS, reason for ICU admission, psychiatric comorbidities, delirium

**Additional file 1: Figure S1. Study Flowchart**


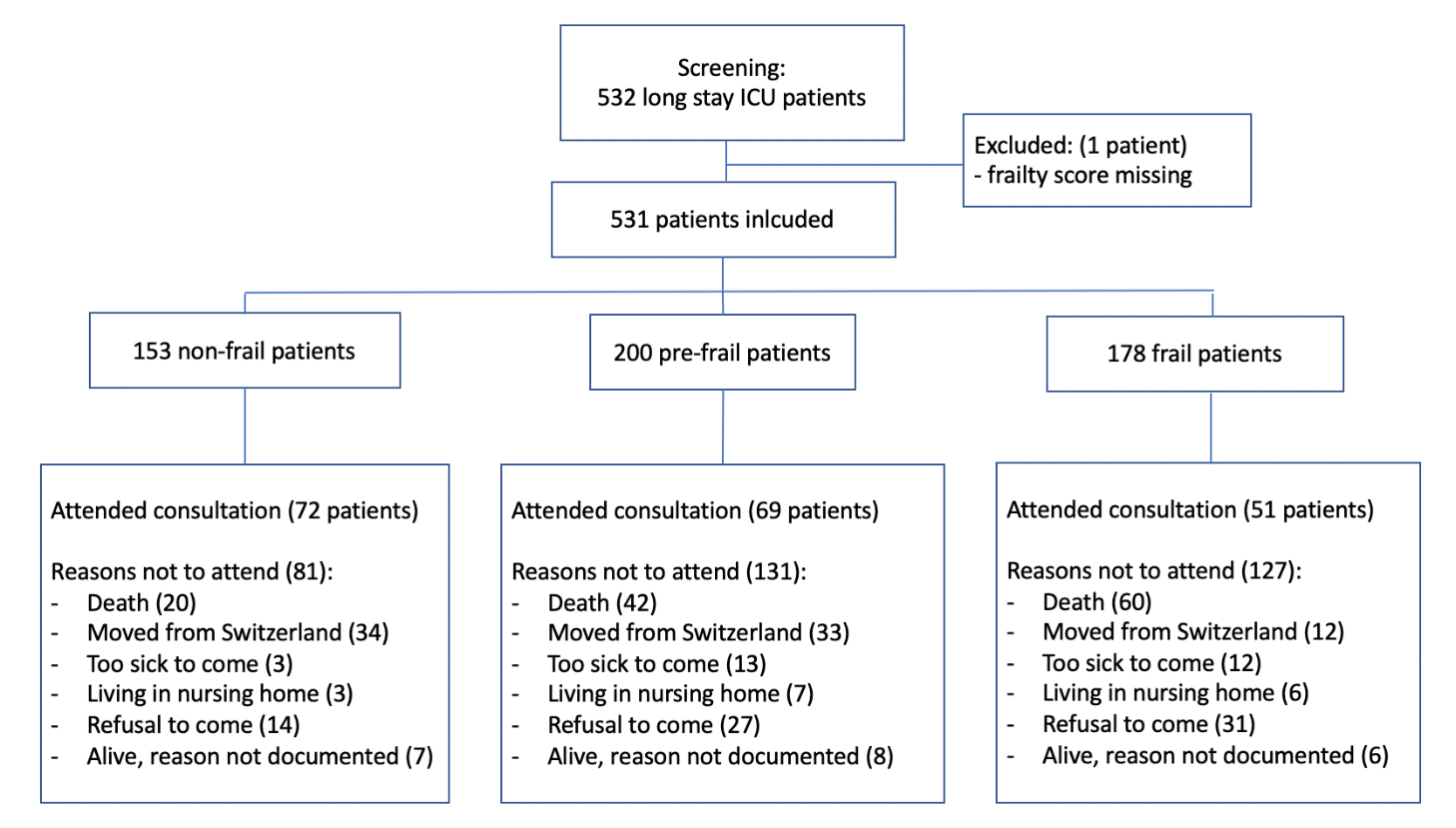

Supplement: Supplementary file 1 — Additional file 1: Table S1. The Clinical Frailty Scale (CFS)–version 1.0. Table S2. Patients follow-up at 6 months according to their frailty score on ICU admission. Table S3. Sensitivity analysis: descriptions of long stay ICU patients according to their frailty score on ICU admission, excluding patients who did not come to the ICU consultation. Table S4. Association Between Frailty and Psychological, Cognitive, and Physical Outcomes at 6 Months after ICU Admission in Surviving ICU Patients. Figure S1. Study Flowchart. [file 13613_2024_1261_MOESM1_ESM.docx]
